# Computed tomography-based coronary lumen volume to myocardial mass ratio in patients undergoing transcatheter aortic valve replacement: a novel method for risk assessment

**DOI:** 10.1186/s12872-025-04705-9

**Published:** 2025-04-24

**Authors:** Wenting Li, Ruichen Ren, Qingyuan Zhao, Chengcheng Qi, Zhiyu Chen, Yang Zhang

**Affiliations:** Department of Radiology, Qilu Hospital of Shandong University, Jinan, Shandong China

**Keywords:** Coronary computed tomography angiography, Transcatheter aortic valve replacement, Coronary lumen volume, Left ventricle myocardial mass, Volume to myocardial mass

## Abstract

**Background:**

The coronary lumen volume to myocardial mass (V/M) ratio has been suggested as a quantitative metric of potential imbalance between coronary blood supply and myocardial oxygen demand. This study was designed to assess the prognostic value of the V/M ratio for predicting major adverse cardiovascular events (MACE) in patients undergoing transcatheter aortic valve replacement (TAVR).

**Methods:**

This study enrolled patients who received a standard planning computed tomography (CT) scan before TAVR and dichotomized at the median of 33.31 mm³/g of V/M ratio into groups with low V/M ratio and high V/M ratio. The V/M ratio was calculated by coronary computed tomography angiography (CTA). The endpoint was a composite of all-cause mortality, stroke, and hospitalization for heart failure. The cumulative incidence of the MACE was compared using Kaplan-Meyer plots and uni- and multivariate Cox proportional hazards regression analysis.

**Results:**

In total, 139 patients were enrolled in this study finally (mean age 71.7 ± 6.7 years, 41.7% female). The mean V/M ratio was considerably lower in patients with MACE than in those without MACE (26.5 ± 4.9mm^3^/g vs. 34.0 ± 3.8mm^3^/g, *P*<0.001). Multivariate Cox proportional hazards regression showed that the low V/M ratio group (≤ 33.31 mm³/g) had a higher risk of MACE after TAVR (HR: 6.14, 95%CI: 1.37–27.54; *P* = 0.018).

**Conclusions:**

The lower V/M ratio could serve as an independent predictor of MACE in patients undergoing TAVR.

**Clinical trial number:**

Not applicable.

**Supplementary Information:**

The online version contains supplementary material available at 10.1186/s12872-025-04705-9.

## Introduction

Aortic stenosis (AS) is the most common valvular heart disease in developed countries and among the aging population [1, 2]. Its typical clinical manifestations are dyspnea, chest pain and syncope. It has been reported that up to 20% of patients with severe AS have acute decompensation [3], including heart failure, cardiogenic shock. For patients with moderate or severe AS who have not received treatment, the 5-year mortality rate is as high as 50% [4,5]. Transcatheter aortic valve replacement (TAVR) is a common treatment option for advanced aortic valve disease and a minimally invasive alternative to traditional surgical aortic valve replacement [6–8].

In patients undergoing TAVR, the coronary artery disease (CAD) often occurs concurrently with severe AS and both have similar risk factors and pathogenesis [9–11]. Patients with progressive AS experience left ventricular hypertrophy (LVH) and impaired coronary flow reserve (CFR), resulting in a mismatch between coronary blood supply and myocardial oxygen demand [12–14], ultimately producing clinically manifest as angina despite normal epicardial coronary arteries [15].

The coronary lumen volume to myocardial mass (V/M) ratio, founded on the principle of allometric scaling laws, was initially described by Gould et al. more than 40 years ago [16]. It is considered a new marker of supply-demand mismatch that emphasizes the linear correlation between coronary lumen volume and myocardial mass [17–19]. Previous studies [20–22]indicated that the Low V/M ratio was linked with severe CAD, decreased myocardial blood flow, and fractional flow reserve (FFR) ≤ 0.80.

The purpose of our study was to assess the prognostic value of CT-derived V/M ratio in patients undergoing TAVR.

## Methods

### Participant population

This study enrolled symptomatic patients with severe aortic stenosis [23] who underwent TAVR between January 2021 and December 2023. All patients who underwent CT scans before TAVR also received coronary CTA examinations at our center. The date of the coronary CTA examination was defined as the study enrollment date (the starting point of the study) for each patient.

Exclusion criteria: (1) History of previous coronary interventions or previous cardiac or valvular surgery; (2) Incomplete clinical data; (3) No preprocedural coronary CTA; (4) Poor image quality, such as images with respiratory or motion artifacts, prevents the artificial intelligence software from conducting effective post-processing analysis, ultimately leading to the failure of V/M ratio calculation; (5) Inconsistent CT scanning parameters; (6) Lost to follow-up. This study complied with the Declaration of Helsinki, was approved by the Research Ethics Committee of Qilu Hospital of Shandong University (approval number: *KYLL-202401-056*). Written informed consent was waived from all participants.

### Imaging protocol

All examinations were conducted with a third-generation dual-source CT scanner (Somatom Force, Siemens Healthineers, Germany). Before the scan, patients were trained for breath holding to reduce respiratory motion artifacts and improve the success rate of the examination. The prospective or retrospective electrocardiogram gating mode was selected based on the patient’s heart rate after breath holding (the acquisition phase was 38-78% of the R-R interval). All patients underwent non-enhanced CT scans before CTA scanning for the quantitative evaluation of coronary artery calcium score (CACS). According to the patient’s vascular and cardiac function, the injection rate of 3.5-5.0 ml/s was selected to inject 30.0–55.0 ml of ionic contrast agent iopromide (containing 370 mgI/ml, Bayer Healthcare, Germany) and 40.0–60.0 ml of 0.9% sodium chloride injection into the median cubital vein. The scan parameters were set as follows: detector collimation 192 × 0.6 mm, slice thickness 0.75 mm, rotation time 0.25 s/r, temporal resolution 75 ms, tube voltage 80–120 kv, and tube current was automatically adjusted according to the patient’s body size by the automatic exposure control system (CARE Dose 4D, Siemens Healthineers, Germany). When evaluating the coronary arteries, the CT system automatically reconstructed the data of the optimal systolic and diastolic phases under a slice thickness of 0.75 mm and the Bv40 convolution kernel.

#### Coronary artery stenosis and calcium score analysis

CACS was calculated using Agatston score and measured by ‘Calcification Score Module’ of Syngo.via software. The degree of coronary lumen diameter stenosis (DS%) was defined as (proximal normal lumen diameter − minimum lumen diameter)/proximal normal lumen diameter*100%. Any coronary segment with a diameter reduction of 50% or greater was classified as having significant stenosis [24].

#### Coronary lumen volume to myocardial mass (V/M) analysis

**Fig. 1:**
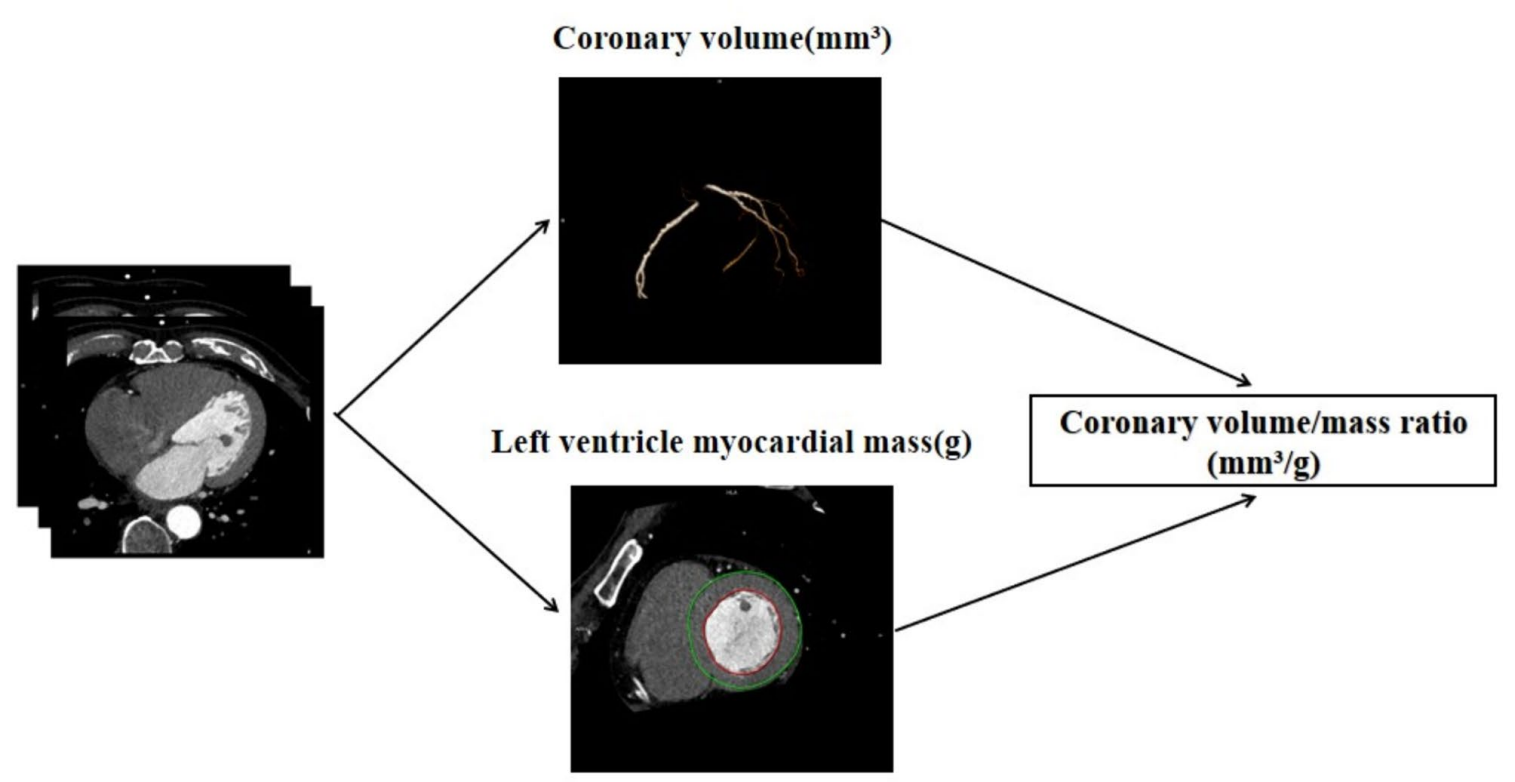
Methodology for computing the V/M ratio. The coronary artery vessel tree was segmented from the coronary CTA dataset and the coronary lumen volume was calculated for all vessels and branches ≥ 1.5 mm in diameter. Left ventricular myocardial mass was extracted from the coronary CTA dataset and computed with dedicated software. Finally, the V/M ratio was computed by dividing coronary lumen volume over Left ventricular myocardial mass.

First, we select the optimal diastolic images of coronary CTA. These images are then opened in the ‘CT Cardiac’ module (Syngo.via, Siemens Healthcare). Once loaded, the workstation initiates the corresponding post-processing procedures for analysis. In the ‘CT Coronary’ sub-module, the system automatically segments and extracts the coronary artery tree, aorta, and myocardium. By applying the Boolean operation, we subtract the aorta from the coronary artery tree to isolate the coronary blood vessels. Subsequently, we adjust the threshold (ranging from 50–665 HU) to display only the vascular tissue structure. After this adjustment, the module automatically calculates the total coronary lumen volume (mm³). Next, we use the ‘CT Cardiac Function’ sub-module. This sub-module automatically segments and extracts the myocardium and calculates the left ventricular mass (g). Finally, we obtain the V/M ratio (mm³/g) by dividing the total coronary volume by the left ventricular mass (as shown in Fig. 1).

### Clinical endpoints and follow-up

Follow-up data were collected and assessed through telephone interviews with trained researchers who were blinded to clinical information. The median follow-up was 443 days (IQR:365-520days). The definition of clinical adverse events after TAVR followed the VARC-3 guidelines published in 2021 by the International Valve Academic Research Consortium [25]. The primary endpoint of this study was defined as composite MACE after TAVR, including all-cause mortality, stroke, and hospitalization for heart failure.

### Statistical analysis

The low and high V/M were defined based on the median V/M in the entire patient cohort. Continuous variables were assessed for normality using the Shapiro-Wilk test. Continuous variables with a normal distribution are presented as mean ± standard deviation (SD) and compared using the Student’s *t test*; Non-normally distributed continuous variables are presented as median with [interquartile range (IQR)] and compared using the Mann-Whitney *U* test. Categorical variables are expressed as frequencies with corresponding percentages [n (%)] and compared using the chi-square test or Fisher’s exact probability method. The cumulative incidence of the MACE was presented as Kaplan-Meier survival curve and compared by a log-rank test. Hazard ratios (HR) and 95% confidence intervals (CI) were calculated according to Cox proportional hazards regression. Parameters significantly associated with the primary outcome (*P*<0.05) were included in the multivariate Cox proportional hazards regression analysis. A *P*-value of <0.05 (two-sided) was considered significant. Statistical analysis was performed using SPSS (version 27.0, SPSS Inc) and R software (version 4.4.0, R Foundation for Statistical Computing).

## Results

### Patient cohort

**Fig. 2:**
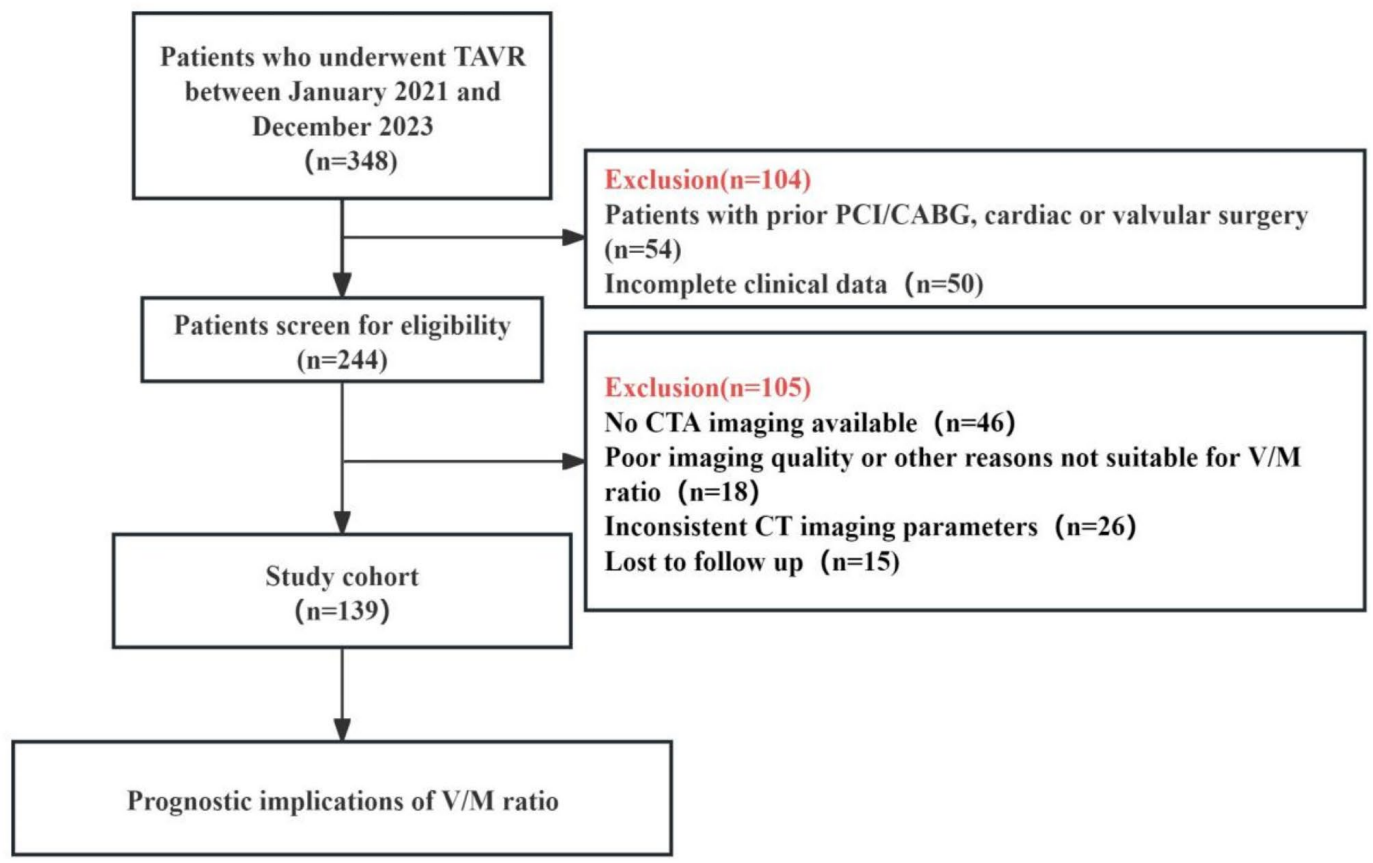
Flowchart of the participant enrollment process. Abbreviations: TAVR = transcatheter aortic valve replacement, PCI = percutaneous coronary intervention, CABG = coronary artery bypass grafting, CTA = computed tomography angiography, V/M = Coronary lumen volume to myocardial mass

A flowchart of the participant enrollment process is displayed in Fig. 2. A total of 139 patients were included in the final analysis of this study. The mean age of this cohort was 71.7 ± 6.7 years, and 58 (41.7%) were female.

### Clinical characteristics

During the median follow up of was 443 days (IQR:365-520days), 16 patients (11.5%) experienced the primary study outcome, including 13 patients with all-cause mortality, 1 patient with stroke, and 2 patients with readmission due to heart failure. Among the patients with all-cause mortality, the most common cause was cardiovascular origin death. Patients were categorized into MACE and non-MACE groups according to the presence of the primary endpoint (Table 1). Differences in chronic kidney disease (CKD), European System for Cardiac Operative Risk Evaluation (EuroSCORE II, details shown in Supplemental Table 1), moderate or severe tricuspid regurgitation and local anesthesia were found in patients with and without MACE events (*P*<0.05).

**Table 1:** Baseline characteristics in patients with and without MACE after TAVR

Participants characteristics (*n* = 139)	non-MACE (*n* = 123)	MACE (*n* = 16)	*P* Value
Age (years)	71.4 ± 6.5	74.0 ± 7.6	0.141^a^
Female	50(40.7)	8(50.0)	0.476^c^
BMI (kg/m2)	24.4 ± 3.5	23.3 ± 3.4	0.249^a^
Clinical characteristics			
Hypertension	61(49.6)	9(56.3)	0.616^c^
Diabetes mellitus	26(21.1)	2(12.5)	0.527^c^
Dyslipidemia	27(22.0)	3(18.8)	1.000^c^
Previous and current smokers	38(30.9)	5(31.3)	1.000^c^
Atrial fibrillation	20(16.3)	5(31.3)	0.166^c^
Previous myocardial infarction	5(4.1)	2(12.5)	0.185^c^
Previous stroke or TIA	9(7.3)	3(18.8)	0.144^c^
CKD (KDIGO grade ≥ 3)	1(0.8)	2(12.5)	**0.035** ^**c**^
NYHA heart failure class ≥ III	69(56.1)	9(56.3)	0.991^c^
EuroSCORE II	2.6(1.8–4.1)	5.5(1.9–9.7)	**0.030** ^**b**^
Echocardiographic findings			
LVEF	60%(40-70%)	40%(30-60%)	0.078^b^
Mitral regurgitation (Moderate or severe)	37(30.1)	6(37.5)	0.572^c^
Tricuspid regurgitation (Moderate or severe)	16(13.0)	6(37.5)	**0.022** ^**c**^
Procedural data			
Anesthesia (Local)	64(52.0)	3(18.8)	**0.012** ^**c**^
Vascular access (Transfemoral)	122(99.2)	15(93.8)	0.218^c^
Implanted valve size (Diameter ≥ 27 mm)	49(39.8)	7(43.8)	0.764^c^
TTE Post-procedural PVL (Mild)	47(38.2)	6(37.5)	0.956^c^

Abbreviations: BMI = body mass index, TIA = transitory ischemic attack, CKD = chronic kidney disease, KDIGO = Kidney Disease: Improving Global Outcomes, NYHA = New York Heart Association, EuroSCORE II = European System for Cardiac Operative Risk Evaluation, LVEF = left ventricular ejection fraction, TEE = transthoracic echocardiography, PVL = paravalvular leak

Data are mean ± standard or n(%), medians(interquartile range)

^a^ Student’s t-test

^b^ Mann–Whitney U test

^c^ Chi-squared test or Fisher’s exact probability method

For coronary CTA parameters (Table 2), a higher proportion of patients in the MACE group had ≥ 50% diameter stenosis (*P*=0.039). Moreover, patients in the MACE group had a higher left ventricle myocardial mass compared to patients in the non-MACE group (235.9 ± 52.6 g vs. 192.6 ± 51.8 g, *P* = 0.002). Still, the coronary lumen volume was comparable between both groups(6231.9 ± 1797.9mm^3^ vs. 6492.4 ± 1721.9mm^3^, *P*=0.509). Finally, for patients in the MACE group, the V/M ratio was considerably lower than in the non-MACE group (26.5 ± 4.9mm^3^/g vs. 34.0 ± 3.8mm^3^/g, *P*<0.001).

**Table 2:** Coronary CTA parameters in patients with and without MACE after TAVR

Participants characteristics (*n* = 139)	non-MACE (*n* = 123)	MACE (*n* = 16)	*P* Value
CACS (Agatston units)	262.5 (30.0-627.6)	613.7 (70.7-1161.7)	0.108^b^
Diameter stenosis (%) ≥ 50%	51 (41.5)	11 (68.8)	**0.039** ^**c**^
Coronary lumen volume (mm^3^)	6492.4 ± 1721.9	6231.9 ± 1797.9	0.509^b^
Left ventricle myocardial mass (g)	192.6 ± 51.8	235.9 ± 52.6	**0.002** ^**a**^
Coronary lumen volume/mass ratio (mm^3^/g)	34.0 ± 3.8	26.5 ± 4.9	**<0.001** ^**b**^

Abbreviations: MACE = major adverse cardiovascular events, CACS = coronary artery calcium score

^a^ Student’s t-test

^b^ Mann–Whitney U test

^c^ Chi-squared test or Fisher’s exact probability method

**Fig. 3:**
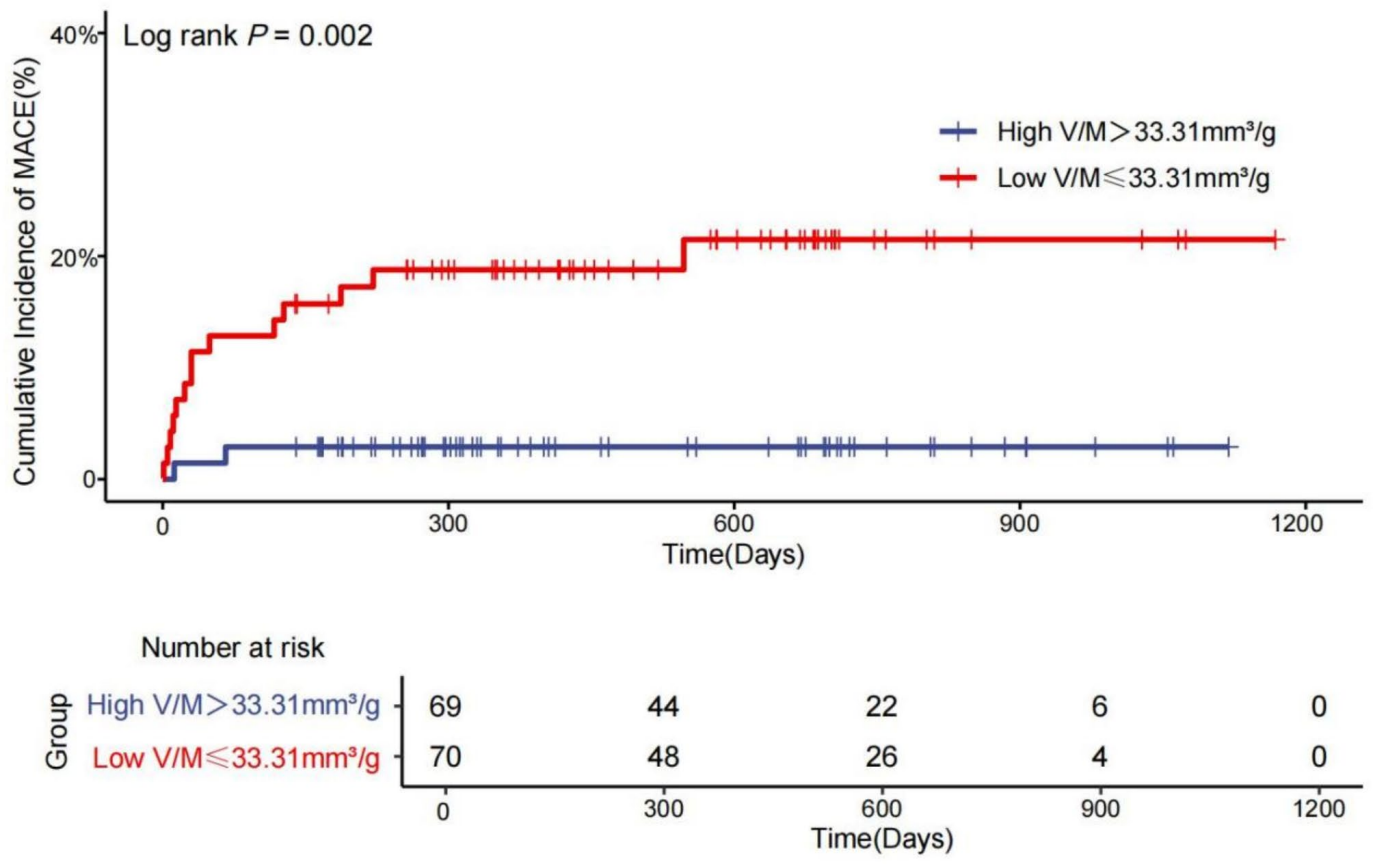
Cumulative incidence of MACE after TAVR intervention based on the V/M ratio. Graphical representation of a Cox proportional hazards model; patients with V/M>33.31 mm³/g depicted via the dark blue line, those with V/M ≤ 33.31 mm³/g in red. Abbreviations: MACE = major adverse cardiovascular events; V/M = Coronary lumen volume to myocardial mass

In univariate Cox regression analysis(Table 3; Fig. 3), V/M ratio ≤ 33.31 mm^3^/g was a predictor of MACE after TAVR (HR:7.27, 95%CI: 1.65–32.01, *P*=0.009). In addition, the presence of CKD (HR:9.09; *P*=0.004), EuroSCORE II (HR:1.26, *P*<0.001), LVEF (HR:0.05, *P*=0.046), tricuspid regurgitation (HR:3.45, *P* = 0.017), DS%≥50% (HR:2.92, *P* = 0.047) and local anesthesia (HR: 0.25, *P* = 0.034)were also associated with increased risk of MACE after TAVR. The results of the univariate Cox regression analysis for other variables were presented in Supplementary Table 2. In multivariate Cox proportional hazards regression (Table 3), V/M ratio ≤ 33.31 mm^3^/g (HR: 6.14, 95%CI: 1.37–27.54; *P* = 0.018) was significantly associated with the incidence of MACE.

**Table 3:** Uni- and multivariate analysis for the prediction of MACE after TAVR

	Univariate		Multivariate	
	HR (95%CI)	*P* Value	HR (95%CI)	*P* Value
CKD (KDIGO grade ≥ 3)	9.09 (2.04–40.57)	**0.004**	1.56 (0.24–10.26)	0.647
EuroSCORE II	1.26 (1.12–1.41)	**<0.001**	1.10 (0.92–1.31)	0.312
LVEF	0.05 (0.00-0.95)	**0.046**	0.71 (0.02–30.77)	0.860
Tricuspid regurgitation (Moderate or severe)	3.45 (1.25–9.50)	**0.017**	2.23 (0.74–6.69)	0.155
Diameter stenosis (%) ≥ 50%	2.92 (1.02–8.42)	**0.047**	2.74 (0.81–9.32)	0.106
V/M ratio ≤ 33.31 (mm^3^/g)	7.27 (1.65–32.01)	**0.009**	6.14 (1.37–27.54)	**0.018**
Anesthesia (Local)	0.25 (0.07–0.90)	**0.034**	0.33 (0.09–1.25)	0.104

Abbreviations: CKD = chronic kidney disease, KDIGO = Kidney Disease: Improving Global Outcomes, LVEF = left ventricular ejection fraction, V/M = Coronary lumen volume/ left ventricle myocardial mass

## Discussion

To our knowledge, this study was the first to determine the V/M ratio in patients undergoing TAVR. Based on the Kaplan–Meier survival curve, the low V/M ratio tended to be associated with a higher cumulative incidence of MACE after TAVR. Multivariate Cox proportional hazards regression analysis revealed that the low V/M ratio was independently associated with the incidence of MACE.

Interestingly, we observed that patients who experienced MACE after TAVR had a higher left ventricular myocardial mass. This increase in mass contributed to a lower V/M ratio, whereas the coronary lumen volume remained relatively stable between the groups. Thus, the elevated left ventricular myocardial mass can account for the difference in the V/M ratio. There are several potential mechanisms that may explain the observed increase in left ventricular mass and the consequent reduction in the V/M ratio among patients in the MACE group.

One possible explanation is that LVH in patients with AS is predominantly an adaptive response to increased left ventricular afterload [26]. However, this adaptive process triggers a series of adverse hemodynamic changes. These include elevated left ventricular cavity pressure [14], reduced coronary perfusion pressure [27], and shortened perfusion times [28]. Ultimately these changes lead to subendocardial ischemia, myocardial apoptosis [29], and fibrosis [30], all of which are detrimental to post - operative cardiovascular outcomes [31].

Renin–angiotensin-aldosterone system (RAAS) inhibitors are thought to be capable of reducing the left ventricular afterload in AS patients, thereby alleviating myocardial hypertrophy and fibrosis [32]. Additionally, RAAS inhibitors can lower blood pressure levels, further reducing the overall hemodynamic burden on the left ventricle of AS patients [33]. Basile et al. found [34] that RAAS inhibitor treatment at baseline was independently associated with a lower risk of 2-year cardiovascular mortality in patients with severe aortic stenosis undergoing TAVR (HR = 0.44, *P* = 0.009). This finding suggests that, in addition to reducing LVH, these inhibitors can improve coronary blood flow. They correct the imbalance between myocardial oxygen supply and demand in AS patients, thereby enhancing the prognosis of those undergoing TAVR. This therapeutic approach appears to hold great promise.

Moreover, early researches [35, 36] suggested that the density of the coronary microvascular bed is diminished in animals with AS, indicating an inadequate growth of new vessels during hypertrophy, which further exacerbates the mismatch between coronary blood supply and myocardial oxygen demand. This could provide a second explanation for the lower V/M ratios observed in patients with the MACE group.

The V/M ratio is a newly available anatomical parameter capable of revealing a potential physiological imbalance between the supply (coronary lumen volume) and demand (myocardial mass). It can be easily quantified from conventional TAVR planning CT without additional diagnostic procedures or radiation exposure. The role of the V/M ratio has been examined in different clinical backgrounds and concerning different risk factors [37–39], such as sex, smoking, hypertension.

Although these cardiovascular risk factors showed no significant differences between the low and high V/M ratio groups in our study(shown in Supplementary Table 3), this may be attributable to differences in patient characteristics and sample size. However, our findings indicate that a lower preoperative baseline V/M ratio for TAVR is associated with a higher risk of adverse outcomes. Thus, these patients should undergo closer monitoring and follow up after the procedure. RAAS inhibitors can be considered for baseline treatment in TAVR candidates to correct the V/M ratio imbalance. Nevertheless, when using these inhibitors, aspects like the timing of administration and dosage need to be carefully considered. Additionally, in the future, incorporating the V/M ratio into the preoperative coronary CTA for TAVR should be taken into account. As such, the V/M ratio has the potential to play a crucial role in the risk stratification of patients after TAVR.

### Study limitations

There are some limitations in our study. Firstly, it is a single-institution study with a relatively small sample size and a low incidence of primary outcome, which may increase the risk of model overfitting, limit our ability to assess prevention strategies, and lead to insufficient statistical validation. Therefore, in this study, we constructed a more concise and targeted prediction model, aiming to reduce the overfitting risk caused by the introduction of too many variables. Future studies should consider increasing the sample size to improve the statistical efficacy and reliability. Multicenter collaborations could also enhance the sample representativeness and the generalizability of the study. Secondly, it is worth noting that the V/M ratio was calculated at the overall patient level. However, we could not exclude the possibility that within a single patient, the V/M ratio might differ in different coronary regions. Finally, the CCTA image data in this study were reconstructed at 75% ± 5% of the RR interval. As a result, the calculated left ventricular mass reflects mid-diastolic mass rather than end-diastolic mass. However, mid-diastolic left ventricular mass has been validated and shows a strong correlation with standard assessments of left ventricular mass [40].

## Conclusions

The V/M ratio could be regarded as a novel non-invasive imaging biomarker, able to provide useful insight into cardiac physiology, which is likely to have promising applications in AS cohorts undergoing TAVR.

## Electronic supplementary material

Below is the link to the electronic supplementary material.

Supplementary Material 1

## Data Availability

No datasets were generated or analysed during the current study.

## Abbreviations

V/M: Coronary lumen volume to myocardial mass
TAVR: Transcatheter aortic valve replacement
MACE: Major adverse cardiovascular events
CT: Computed tomography
CTA: Computed tomography angiography
AS: Aortic stenosis
CAD: Coronary artery disease
LVH: Left ventricular hypertrophy
CFR: Coronary flow reserve
BMI: Body mass index
CACS: Coronary artery calcification score
DS%: Diameter stenosis
EuroSCORE II: European System for Cardiac Operative Risk Evaluation II
LVEF: Left ventricular ejection fraction
HR: Hazard ratios
95%CI: 95% confidence intervals
