# Supplementary material for: Computed tomography-based coronary lumen volume to myocardial mass ratio in patients undergoing transcatheter aortic valve replacement: a novel method for risk assessment
Source: BMC Cardiovasc Disord. 2025 Apr 24;25:311. doi: 10.1186/s12872-025-04705-9 (PMC12020294; doi:10.1186/s12872-025-04705-9)
Supplement: Supplementary file 1 — Supplementary Material 1 [file 12872_2025_4705_MOESM1_ESM.docx]

**Supplemental Data***.*

**Supplemental** **Table 1.**

**The EuroSCORE II is calculated according to a previously developed logistic risk model and includes 18 different factors** (**<http://euroscore.org/calc.html>**).

| Patient-related factors (n = 10) | Cardiac-related factors (n = 5) | Operation-related factors (n = 3) |
| --- | --- | --- |
| Age | CCS angina class 4 | Surgery on thoracic aorta |
| biological sex | LV function (ejection fraction) | Urgency of operation |
| Chronic lung disease | Recent myocardial infarction | Weight of operation |
| Extracardiac arteriopathy | Pulmonary hypertension |  |
| Poor mobility | NYHA stage |  |
| Previous cardiac surgery |  |  |
| Active endocarditis |  |  |
| Critical preoperative state |  |  |
| Renal impairment |  |  |
| Diabetes on insulin |  |  |

EuroSCORE is a scoring system which predicts the risk of death for patients considering heart surgery.

Abbreviations: EuroSCORE=European System for Cardiac Operative Risk Evaluation, CCS=Canadian cardiovascular society, LV= Left ventricular, NYHA= New York Heart Association.

**Supplemental** **Table 2. Univariate analysis for the prediction of MACE after TAVR.**

| **Participants characteristics (n = 139)** | **Univariate** | | |
| --- | --- | --- | --- |
|  | **HR (95%CI)** | **P Value** | |
| Age (years) | 1.06 (0.98-1.14) | 0.131 | |
| Female | 1.44 (0.54-3.84) | 0.467 | |
| BMI (kg/m2) | 0.92 (0.80-1.06) | 0.251 | |
| Clinical characteristics | | |  |
| Hypertension | 1.27 (0.47-3.41) | 0.634 | |
| Diabetes mellitus | 0.57 (0.13-2.51) | 0.458 | |
| Dyslipidemia | 0.85 (0.24-2.97) | 0.794 | |
| Previous and current smokers | 1.00 (0.35-2.88) | 0.997 | |
| Atrial fibrillation | 2.19 (0.76-6.31) | 0.146 | |
| Previous myocardial infarction | 3.30 (0.75-14.53) | 0.114 | |
| Previous stroke or TIA | 2.59 (0.74-9.08) | 0.138 | |
| CKD (KDIGO grade≥3) | 9.09 (2.04-40.57) | **0.004** | |
| NYHA heart failure class≥III | 1.03 (0.38-2.77) | 0.952 | |
| EuroSCORE II | 1.26 (1.12-1.41) | **＜0.001** | |
| Echocardiographic findings | | |  |
| LVEF (%) | 0.05 (0.00-0.95) | **0.046** | |
| Mitral regurgitation  (Moderate or severe) | 1.34 (0.49-3.68) | 0.572 | |
| Tricuspid regurgitation  (Moderate or severe) | 3.45 (1.25-9.50) | **0.017** | |
| CCTA parameters | | |  |
| CACS (Agatston units) | 1.00 (1.00-1.00) | 0.270 | |
| Diameter stenosis (%)≥50% | 2.92 (1.02-8.42) | **0.047** | |
| V/M≤33.31 (mm^3^/g) | 7.27 (1.65-32.01) | **0.009** | |
| Anesthesia (Local) | 0.25 (0.07-0.90) | **0.034** | |
| Vascular access (Transfemoral) | 0.14 (0.02-1.09) | 0.061 | |
| Implanted valve size  (Diameter≥27 mm) | 1.18 (0.44-3.17) | 0.740 | |
| TTE Post-procedural PVL (Mild) | 0.97 (0.35-2.68) | 0.958 | |

Abbreviations: BMI=body mass index, TIA=transitory ischemic attack, CKD=chronic kidney disease,

KDIGO=Kidney Disease: Improving Global Outcomes, NYHA=New York Heart Association, EuroSCORE II= European System for Cardiac Operative Risk Evaluation, LVEF=left ventricular ejection fraction, CCTA=coronary computed tomography angiography, CACS=coronary artery calcium score, V/M=Coronary lumen volume to myocardial mass, TEE=transthoracic echocardiography, PVL=paravalvular leak.

# **Supplemental Table 3. Baseline characteristics of patients with Low and High V/M ratio.**

| **Participants characteristics**  **(n = 139)** | **Low V/M ratio**  **(n=70)** | **High V/M ratio**  **(n=69)** | **P Value** |
| --- | --- | --- | --- |
| Age (years) | 71.9±7.0 | 71.5±6.3 | 0.808^b^ |
| Female | 33(47.1) | 25(36.2) | 0.192^c^ |
| BMI (kg/m2) | 24.1±3.5 | 24.4±3.6 | 0.642^a^ |
| Clinical characteristics | | | |
| Hypertension | 34(48.6) | 36(52.2) | 0.671^c^ |
| Diabetes mellitus | 14(20.0) | 14(20.3) | 0.966^c^ |
| Dyslipidemia | 19(27.1) | 11(15.9) | 0.109^c^ |
| Previous and current smokers | 24(34.3) | 19(27.5) | 0.389^c^ |
| Atrial fibrillation | 14(20.0) | 11(15.9) | 0.533^c^ |
| Previous myocardial infarction | 4(5.7) | 3(4.3) | 1.000^c^ |
| Previous stroke or TIA | 8(11.4) | 4(5.8) | 0.237^c^ |
| CKD (KDIGO grade≥3) | 2(2.9) | 1(1.4) | 1.000^c^ |
| NYHA heart failure class≥III | 40(57.1) | 38(55.1) | 0.806^c^ |
| EuroSCORE II | 2.6(1.9-4.9) | 2.7(1.9-4.1) | 0.651^b^ |
| Echocardiographic findings | | | |
| LVEF | 49%(31%-65%) | 60%(37%-67%) | 0.105^b^ |
| Mitral regurgitation  (Moderate or severe) | 22(31.4) | 21(30.4) | 0.899^c^ |
| Tricuspid regurgitation  (Moderate or severe) | 14(20.0) | 8(11.6) | 0.175^c^ |
| CCTA parameters | | | |
| CACS (Agatston units) | 206.3(11.4-636.9) | 326.7(69.4-873.2) | 0.282^c^ |
| Diameter stenosis (%) ≥50% | 30(42.9) | 32(46.4) | 0.676^c^ |
| Procedural data |  |  |  |
| Anesthesia (Local) | 24 (34.3) | 43 (62.3) | **＜0.001^c^** |
| Vascular access (Transfemoral) | 69 (98.6) | 68 (98.6) | 1.000^c^ |
| Implanted valve size  (Diameter≥27 mm) | 31 (44.3) | 25 (36.2) | 0.333^c^ |
| TTE Post-procedural PVL (Mild) | 29 (41.4) | 24 (34.8) | 0.420^c^ |

Abbreviations: BMI=body mass index, TIA=transitory ischemic attack, CKD=chronic kidney disease, KDIGO=Kidney Disease: Improving Global Outcomes, NYHA=New York Heart Association, EuroSCORE II= European System for Cardiac Operative Risk Evaluation, LVEF= left ventricular ejection fraction, CCTA=coronary computed tomography angiography, CACS=coronary artery calcium score, V/M= Coronary lumen volume/myocardial mass, TEE=transthoracic echocardiography, PVL=paravalvular leak.

Data are mean ± standard or n(%), medians(interquartile range).

^a^ Student’s t-test

^b^ Mann–Whitney U test

^c^ Chi-squared test or Fisher's exact probability method
